# Spontaneous spinal epidural hematoma (SSEH) after cesarean section under epidural anesthesia: A case report

**DOI:** 10.1016/j.heliyon.2023.e22855

**Published:** 2023-11-25

**Authors:** Hua Wu, Xuezhu Huang

**Affiliations:** Department of Anesthesiology, Women and Children's Hospital of Chongqing Medical University (Chongqing Health Center for Women and Children), Chongqing, China

**Keywords:** Cesarean section, Case report, Epidural anesthesia, Labor analgesia, Spinal epidural hematoma

## Abstract

Spontaneous spinal epidural hematoma (SSEH) is an uncommon condition that can lead to severe neurological injuries, often accompanied by back pain. Pregnancy is identified as a risk factor for SSEH. Early diagnosis of SSEH presents challenges due to its atypical manifestations and the use of intraspinal anesthesia and analgesic techniques. In this case, we present the instance of a 29-year-old woman who initially received epidural labor analgesia during the first stage of labor but subsequently required a cesarean section under epidural anesthesia according to amniotic fluid turbidity. Unfortunately, the anomalous recovery of neurological function in her left lower extremity was not given sufficient attention at an early stage, and paralysis in the non-puncture segment occurred 45.5 hours after the initial puncture. Interestingly, she did not experience any back pain during these procedures. MRI examination and consultation with neurosurgeons confirmed the diagnosis of SSEH, prompting the patient to undergo emergency decompression surgery. She made an incomplete recovery 17 months after the operation. This case emphasizes the importance of considering the possibility of SSEH in pregnant women undergoing epidural analgesia, highlighting the need for spinal imaging and early neurosurgical interventions to facilitate treatment.

## 1 Introduction

Spontaneous spinal epidural hematoma (SSEH) is an exceedingly rare condition, with an estimated annual incidence of approximately 0.1 per 100,000 in adults [1]. SSEH is characterized by its acute onset, potentially leading to catastrophic compression of neural tissue, either through direct injury or ischemia [2], necessitating immediate surgical intervention upon the emergence of neurological dysfunction [3]. Various gestational complications, including thrombocytopenia or preeclampsia [2], and the epidural vascular dilation [4], can amplify the risk of SSEH. Furthermore, the use of intraspinal anesthesia and analgesic techniques can obscure neurological manifestations, thereby complicating early diagnosis [5]. In this report, we present the case of a pregnant woman who initially received epidural labor analgesia and subsequently underwent an emergency cesarean section under epidural anesthesia. Remarkably, SSEH developed after the surgery, even in the absence of spinal pain.

## 2 Case presentation

**Fig. 1:**
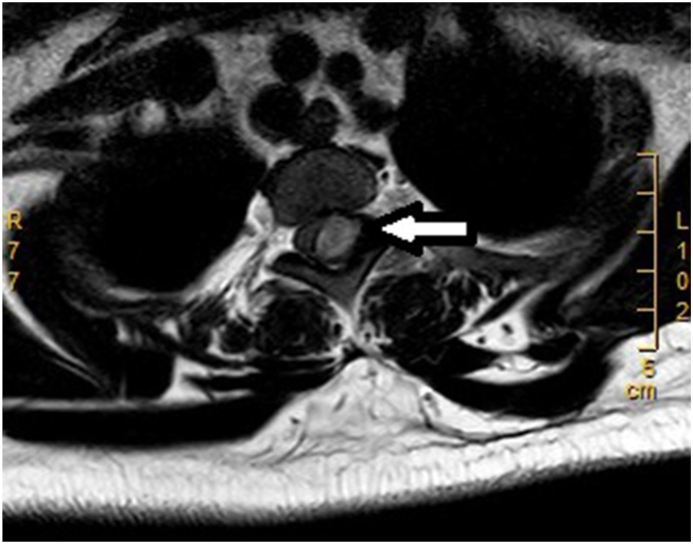
Thoracic MRI of the patient. Axial T3 sequence shows abnormally enhanced epidural signals (arrow), which pushed the spine to the right.

**Fig. 2:**
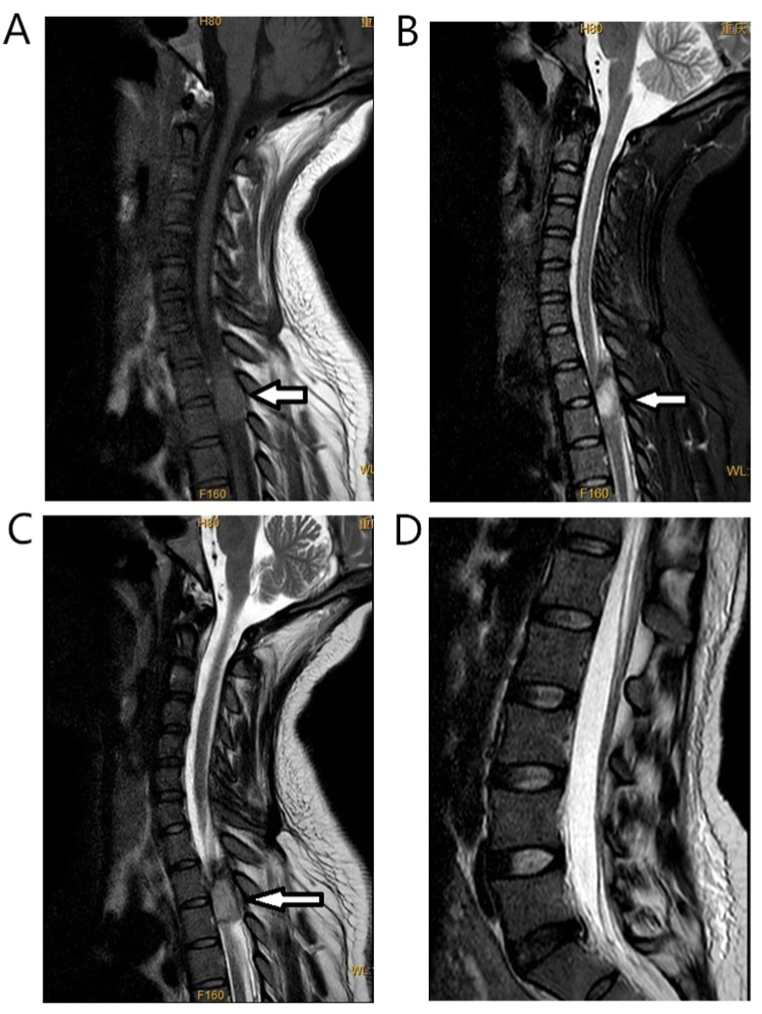
MRI of cervical, thoracic and lumbar vertebrae. Sagittal T2-3 sequence shows epidural abnormality, T1WI and T2FS show high signals (arrows in Fig. 2A and B), and T2WI shows mixed high/low signals (arrow in Fig. 2C). These findings are in agreement with the manifestations of acute epidural hemorrhage. No abnormality was found at the site of lumbar puncture (Fig. 2D).

**Fig. 3:**
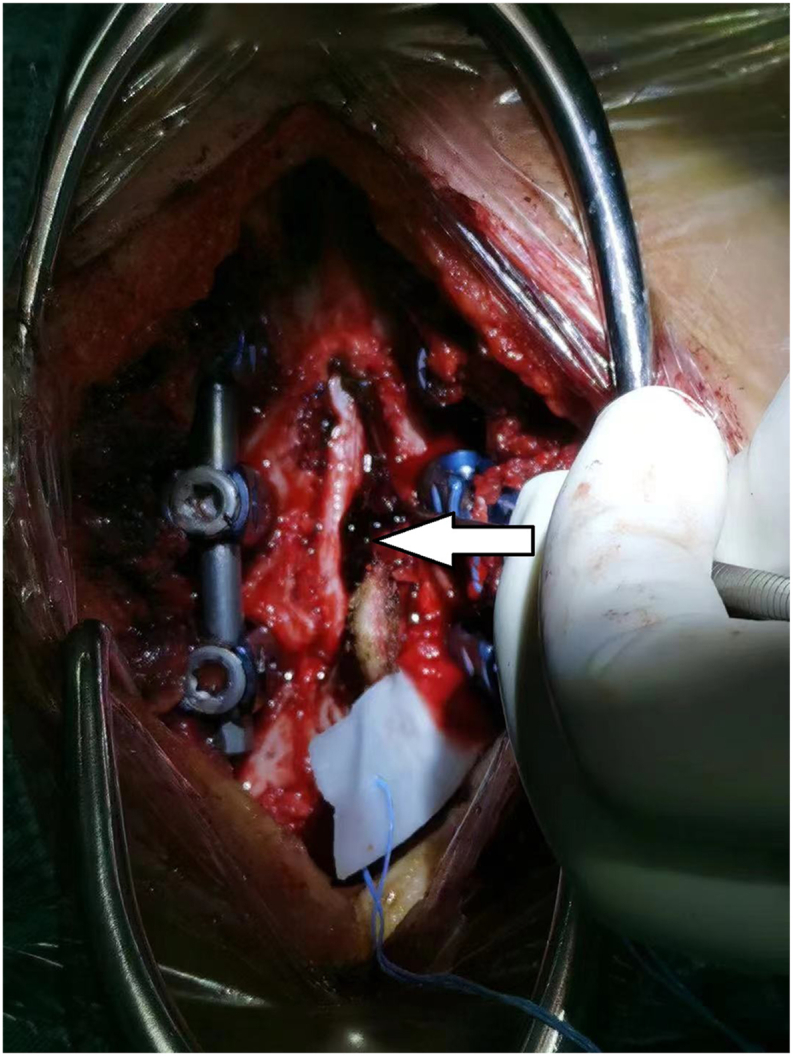
Intraoperative findings show the blood clots (arrow) at the T2 and T3 levels, which pushed the spinal dura to the right.

A 29-year-old primigravida woman had no history of gestational diabetes mellitus, preeclampsia, or coagulation disorders, and no anti-coagulation therapy with heparin. Prenatal laboratory testing showed normal results: platelet count, 157 × 10^9^/L; hemoglobin (Hb), 132 g/L; prothrombin (PT), 10 s; activated partial thromboplastin time (APTT), 27 s; international normalized ratio (INR), 0.81; alanine aminotransferase (ALT), 31 U/L; aspartate aminotransferase (AST), 36U/L; total bile acid (TBA), 9.1 μmol/L. She was scheduled for transvaginal delivery after pregnancy for 41 + 2 weeks on June 3, 2021. She received epidural labor analgesia when the cervix dilated to 2 cm with numerical rating scale (NRS) for pain was 9 (0 represents “no pain” and 10 represents “worst pain imaginable”) [6]: A selective epidural puncture was performed at the L2-3 level, and a 4 cm catheter was inserted and securely fixed. Following the administration of a 4 mL test dose of 2 % lidocaine, a 5 mL epidural solution (comprising ropivacaine 0.125 % and sufentanil 3 μg) was delivered through the catheter. Subsequently, the patient-controlled epidural analgesia (PCEA) pump was connected to the catheter, infusing a mixture of 0.75 % ropivacaine (10 mL) and sufentanil (20 μg) in saline, resulting in a 60 mL solution. The continuous background infusion rate was set at 8 mL per hour, with a patient-controlled dose of 3 mL and a locking interval of 15 minutes.The NRS for pain decreased to 4 following analgesia, with no motor blockade or breakthrough pain observed. Subsequently, 3 h later, the patient underwent an emergency cesarean section due to grade 2 amniotic fluid turbidity. Anesthesia for the cesarean section was administered via the pre-implanted epidural catheter, utilizing 12 mL of 3 % chloroprocaine. The intraoperative blood loss amounted to 400 mL. Post-surgery, patient-controlled intravenous analgesia (PCIA) was initiated. This involved a solution composed of 800 mg tramadol, 0.2 mg fentanyl, and 10 mg dexamethasone dissolved in saline, resulting in a 100 mL mixture. The PCIA regimen featured a background dose of 2 mL per hour, a patient-controlled dose of 0.5 mL, and a 15-min locking interval. The sensation and muscle strength in the right lower limb fully recovered within 15 hours following the puncture. Conversely, mild soreness and swelling were noted in the left lower limb, accompanied by a manual muscle testing (MMT) grade of 2 [7]. Hypesthesia, manifesting below the chest level, abruptly manifested 45.5 hours following the puncture. Subsequently, there was a reduction in the muscle strength of both lower limbs, rapidly progressing to flaccid paralysis. A spinal MRI examination revealed a space-occupying lesion at the left epidural site of T2-3, measuring approximately 12 × 10 × 30 mm. The axial view showed spinal compression with a rightward shift (Fig. 1). The T1WI and T2FS sequences exhibited high signals (Fig. 2A and B), while the T2WI displayed mixed high/low signals (Fig. 2C). Notably, no abnormalities were detected at the lumbar puncture site (Fig. 2D). Following consultations with anesthetists and neurosurgeons, a diagnosis of SSEH was suspected. The patient subsequently underwent laminectomy 14.5 hours after paralysis onset. Intraoperative observations revealed a blood clot on the left side posterior to the T2-T3 vertebral bodies, pushing the spinal dura to the right (Fig. 3). Additionally, it was noted that the posterior veins of the T3 vertebrae and the venous plexus of the intervertebral foramen displayed a cirsoid and circuitous pattern, with thinner venous walls than usual. Pathological examination confirmed the presence of a vascular malformation. Post-laminectomy, the patient received a treatment regimen consisting of methylprednisolone, mannitol, hyperbaric oxygen therapy and aggressive rehabilitation programs, aimed at facilitating the recovery of neurological function. During the last follow-up visit, conducted 17 months post-operation, sensory function in both lower limbs and muscle strength in the right lower limb had been fully restored to normal levels, while the left lower limb achieved a MMT grade of 4. The patient provided written informed consent for the publication of this case report and any accompanying images.

## 3 Discussion

SSEH is an exceedingly rare yet debilitating complication, with risk factors encompassing coagulation dysfunction, vascular deformities, and pregnancy [8]. In this case, we chronicle the experience of a woman who underwent a cesarean section while receiving continuous epidural analgesia and anesthesia. Approximately two days after the initial puncture, she manifested flaccid paralysis. Both the MRI examination and intraoperative observations confirmed the presence of SSEH in non-puncture segments. Neurological symptoms can be easily overlooked, in part due to the effects of anesthesia and the challenge posed by the absence of neurosurgeons, rendering early diagnosis a formidable task [9].

In this case, both intraoperative observations and the pathological examination concurred in identifying a vascular malformation. The venous plexus within the epidural space is noteworthy for its lack of venous valves, rendering it susceptible to variations in intra-thoracic or intra-abdominal pressure [8]. As pregnancy advances, intra-abdominal pressure gradually escalates, consequently elevating the risk of epidural bleeding. Moreover, during uterine contractions, the pressure within the epidural space experiences further augmentation [10]. Notably, the patient underwent an attempted transvaginal delivery, encountering uterine contractions in the process. These physiological changes may have contributed to the onset of SSEH.

While injuries resulting from epidural puncture are recognized as direct causes of secondary epidural hematoma [3,9], the hematoma in this case was located relatively distant from the puncture site, and MRI did not reveal any abnormalities at the site of puncture. We postulate that the epidural puncture itself was not the primary instigator of the epidural hematoma in this instance. However, it remains plausible that the entire epidural procedure, encompassing both the puncture and the administration of local anesthetics, may elevate the risk of epidural hematoma by increasing epidural pressure [10]. In situations where patients who have undergone intraspinal anesthesia develop sudden postoperative neurological dysfunctions, anesthetists typically focus on ascertaining the presence of hematoma at the puncture site within the spinal canal. Yet, if the segment of the spine experiencing sensory deficits lies above the segment where the puncture was performed, it can present a conundrum for anesthetists, especially in the absence of neurosurgeons, potentially leading to delayed diagnosis. Following this case, our medical staff underwent comprehensive training in SSEH management, with a particular emphasis on promptly applying spinal MRI and seeking consultation from the neurosurgery department.

In this case, the bleeding site was observed in the posterior region of the thoracic spinal cord. Previous studies have consistently reported the frequent occurrence of SSEH in the thoracic or thoracolumbar junction [4,8]，which may be explained by this area being the only area of the spinal canal with a compact continuous venous network in the epidural space [3,4]. An anatomical dissection of human cadaver vertebral venous systems revealed a notably greater extent of the thoracic posterior internal vertebral venous plexus in comparison to other segments [11]. This finding may underlie the heightened propensity for bleeding in the posterior aspect of the thoracic spine, a pattern consistent with the findings in our case.

After 15 hours of anesthesia, the left lower limb exhibited mild soreness and swelling, accompanied by a MMT grade of 2, while the right limb fully recovered. Clinicians neglected the protracted and uneven restoration of neurological function, possibly due to an overall positive trend in recovery and longer neurological recovery were not rare in the pregnant women [12,13]. In hindsight, it appears plausible to deduce that the left lower extremity's paralysis at 15 hours postoperatively may have been an early indication of epidural hematoma [8]. Regrettably, this went unnoticed initially. Consequently, this case underscores the importance of maintaining constant vigilance for SSEH when there are prolonged or notably asymmetrical neurological recoveries in both lower extremities.

SSEH typically presents with acute spinal pain, affecting roughly 84.8 % of cases [3]. Nonetheless, achieving a confirmed diagnosis can be notably challenging in patients with concurrent conditions such as cerebral ischemia [14] or cardiac ischemia [15]. A relatively small fraction, approximately 8.6 % [16], experience SSEH without the hallmark spinal pain, and this painless presentation can hinder diagnosis. Our patient similarly did not report back pain; instead, she exhibited mild soreness and swelling in her left lower limb. This discrepancy could be attributed to the alleviating effects of postoperative analgesia [8]. In cases involving patients with severe back pain or lower limb pain, physicians should contemplate the possibility of seeking emergency consultations with neurosurgeons, even when the clinical manifestations are atypical or there is an absence of mobility disorders.

Prompt laminectomy and hematoma removal constitute the primary approach for enhancing the prognosis of patients with SSEH and neurologic deficits [3,4], Additionally, conservative management has yielded favorable results for patients without paralysis in several reported cases [3,17]. The most influential prognostic factors are the initial deficit severity and the time elapsed between symptom onset and surgical intervention [18]. Presently, a debate surrounds the optimal time frame: Certain studies [2,3] suggest a critical window of 12 hours for achieving complete recovery, while McQuarrie et al. [19] advocate extending it to 36 hours. An extensive literature review, encompassing 166 spinal hematoma cases, revealed that patients with motor deficits due to an epidural hematoma, when treated within 12 hours of symptom onset, achieved the highest rate of complete recovery (63.4 %), and 50 % of patients treated more than 24 hours after symptom onset achieved favorable outcomes [20]. In our case, the surgical intervention occurred 14.5 hours after the onset of symptoms in the right lower extremity and 45 hours after the onset in the left lower extremity. The variance in the time interval may have contributed to the complete recovery in the right lower extremity and residual muscle weakness in the left lower extremity. It is also plausible that this outcome is partially attributed to the hematoma's location on the left side, where the compression of neural tissue was more pronounced ipsilaterally.

Despite the delayed diagnosis, the patient exhibited a more favorable postoperative recovery in their lower limbs than originally expected. Furthermore, several other studies have reported that over half of the patients treated between 25 and 48 hours after the onset of symptoms achieved complete recovery or presented only mild neurological deficits [3,20]. This favorable outcome may be attributed to a smaller extent of spinal hematoma (as the involvement of more spinal segments correlates with a worse prognosis) [21], younger age [22], early glucocortieoid therapy [23] and the implementation of intensive rehabilitation programs post-surgery [24].

This report has several limitations. It pertains to an unusual and unique case, making it ethically unfeasible to conduct comparative trials. Furthermore, the follow-up evaluation was limited to a 17-month period post-surgery, thus preventing an assessment of the patient's longer-term neurological recovery.

## 4 Conclusion

SSEH is an infrequent condition associated with the potential for severe consequences. Risk factors include vascular malformation, pregnancy, labor, and epidural procedures. Initial symptoms of SSEH may manifest as extremity pain, asymmetrical or prolonged recovery of neurological function in both lower extremities. Timely spinal imaging assessments and neurosurgical interventions are of paramount importance, particularly in cases involving painless neurological dysfunctions. Given that the time elapsed between symptom onset and surgery is one of the most reliable prognostic indicators, surgical intervention should be expedited for SSEH patients experiencing paralysis.

## Ethics approval and consent to participate

All methods were performed in accordance with the relevant guidelines and regulations. Written informed consent was obtained from the patient.

## Consent for publication

Written informed consent was obtained from the patient for publication of this Case report and any accompanying images. A copy of the written consent is available for review by the Editor of this journal.

## Data availability statement

All data are included in this article.

## Funding

This study was supported by the Beijing Health Alliance Charitable Foundation: NO. KM-20230601-01. The funders had no role in study design, data collection and analysis, decision to publish, or preparation of the manuscript.

## Declaration of generative AI in scientific writing

The authors declare that they did not use AI or AI technologies in the writing process.

## CRediT authorship contribution statement

**Hua Wu:** Writing – original draft, Conceptualization. **Xuezhu Huang:** Writing – review & editing, Writing – original draft, Data curation, Conceptualization.

## Declaration of competing interest

The authors declare that they have no known competing financial interests or personal relationships that could have appeared to influence the work reported in this paper.
